# Spared Primary Motor Cortex and The Presence of MEP in Cerebral Palsy Dictate the Responsiveness to tDCS during Gait Training

**DOI:** 10.3389/fnhum.2016.00361

**Published:** 2016-07-19

**Authors:** Luanda A. Collange Grecco, Claudia Santos Oliveira, Manuela Galli, Camila Cosmo, Natália de Almeida Carvalho Duarte, Nelci Zanon, Dylan J. Edwards, Felipe Fregni

**Affiliations:** ^1^Center of Pediatric Neurosurgery, CENEPE-RehabilitationSão Paulo, Brazil; ^2^Movement Analysis Laboratory, Department of Rehabilitation Sciences, University Nove de JulhoSão Paulo, Brazil; ^3^Department of Electronics, Computer Science and Bioengineering (DEIB), Politecnico di MilanoMilano, Italy; ^4^IRCCS San Raffaele PisanaRome, Italy; ^5^PostGraduate Program—Interactive Process of Organs and Systems, Health Sciences Institute, Federal University of BahiaSalvador, Brazil; ^6^Departments of Neurology and Neuroscience, Burke Medical Research Institute, Weill Medical College of Cornel UniversityWhite Plains, NY, USA; ^7^Department of Physical Medicine and Rehabilitation, Spaulding Rehabilitation Hospital, Massachusetts General Hospital, Harvard Medical SchoolBoston, MA, USA

**Keywords:** cerebral palsy, neuromodulation, non-invasive brain stimulation, gait training, motor evoked potential

## Abstract

The current priority of investigations involving transcranial direct current stimulation (tDCS) and neurorehabilitation is to identify biomarkers associated with the positive results of the interventions such that respondent and non-respondent patients can be identified in the early phases of treatment. The aims were to determine whether: (1) present motor evoked potential (MEP); and (2) injuries involving the primary motor cortex, are associated with tDCS-enhancement in functional outcome following gait training in children with cerebral palsy (CP). We reviewed the data from our parallel, randomized, sham-controlled, double-blind studies. Fifty-six children with spastic CP received gait training (either treadmill training or virtual reality training) and tDCS (active or sham). Univariate and multivariate logistic regression analyses were employed to identify clinical, neurophysiologic and neuroanatomic predictors associated with the responsiveness to treatment with tDCS. MEP presence during the initial evaluation and the subcortical injury were associated with positive effects in the functional results. The logistic regression revealed that present MEP was a significant predictor for the six-minute walk test (6MWT; *p* = 0.003) and gait speed (*p* = 0.028), whereas the subcortical injury was a significant predictor of gait kinematics (*p* = 0.013) and gross motor function (*p* = 0.021). In this preliminary study involving children with CP, two important prediction factors of good responses to anodal tDCS combined with gait training were identified. Apparently, MEP (integrity of the corticospinal tract) and subcortical location of the brain injury exerted different influences on aspects related to gait, such as velocity and kinematics.

## Introduction

The neuromodulatory effects of transcranial direct current stimulation (tDCS) on cortical excitability can optimize motor learning and functional improvements in patients with neurological injuries (Lefebvre et al., 2015), which is a promising therapeutic technique for gait rehabilitation (Jayaram and Stinear, 2009; Tahtis et al., 2014). Previous studies involving children with cerebral palsy (CP) have found that anodal tDCS over the primary motor cortex combined with gait training resulted in positive effects in comparison to sham stimulation (Duarte et al., 2014; Grecco et al., 2014, 2015). However, a detailed analysis (group vs. individual effects) of the our data revealed varied effects.

Although the effect of tDCS has been well-documented and seems to be a promising therapeutic tool for this neurological disorder, there are as-yet no clear predictors of responsiveness. Based on the experience of our research group, some children achieved excellent results following anodal tDCS, whereas others demonstrated no effect. Based on the heterogeneity of our discoveries, we believe that tDCS would be effective for patients in which the brain injury spared the motor cortex (cortical vs. subcortical lesions) under the stimulating electrode. We also believe that children with a corticospinal pathway responsive to transcranial magnetic stimulation (TMS), as indicated by the presence of the motor-evoked potential (MEP) in the quadriceps muscle, would have a better response to tDCS. This suggests the potential of the susceptibility of areas of corticospinal system responsible for the motor control of the lower limbs, specifically the quadriceps muscle, to activation by local electrical fields.

There is no complete understanding of how neuroimaging and TMS findings are related to motor function or whether a given patient will respond well when noninvasive brain stimulation is combined with physical therapy, thereby benefiting the motor rehabilitation process. Identifying respondent and non-respondent patients is a major focus of studies involving brain stimulation intervention. Knowledge of neurophysiologic and neuroanatomic predictors of the responsiveness to tDCS is critical in the context of clinical research and it is especially important in guiding the choice of an effective intervention (Brunoni et al., 2015) for a given child.

In the present study, we investigated clinical and neurophysiologic variables to test whether age, gross motor function, laterality of motor impairment (hemiparesis or diparesis), injury location (cortical or subcortical) and MEP (present or absent) are predictors of the responsiveness to tDCS over the primary cortex associated with gait training for children with CP. The secondary aim was to compare the effects of the MEP and injury location, considering active and sham tDCS intervention in the six-minute walk test (6MWT), gait speed, kinematic gait profile and gross motor function in children with CP. Our hypothesis was that children with cortical injuries would respond less to tDCS intervention than those with a subcortical injury. We also predicted that the presence of MEP would favor responsiveness to tDCS, as it demonstrates local susceptibility to electric fields.We further explored the data for an interaction among neurophysiologic and neuroanatomic variables during tDCS and the effects on gait function.

## Materials and Methods

In the present study, we evaluated data from our three previous trials to analyze response predictors of anodal tDCS regarding gait performance (Duarte et al., 2014; Grecco et al., 2014, 2015). These studies received approval from the Human Research Ethics Committee of Universidade Nove de Julho, Brazil, under process number 69803/2012 and was conducted in accordance with the ethical standards laid down in the 1964 Declaration of Helsinki. Each study was parallel, randomized, sham-controlled and double-blinded. The eligibility criteria of our previous studies were a diagnosis of spastic hemiparetic or diparetic CP, children with cortical or subcortical lesions, classification on levels I, II or III of the gross motor function classification (Palisano et al., 1997), independent gait for at least 12 months, age between 5 and 10 years, and degree of comprehension compatible with the execution of the procedures. The exclusion criteria were a history of surgery or neurolytic block in the previous 12 months, orthopedic deformities, epilepsy, metal implants in the skull, the use of hearing aids, anticonvulsant or muscle relaxing drugs. In these protocols, children were randomly allocated to two treatment groups: (1) treadmill training combined with anodal or sham tDCS (Grecco et al., 2014); and (2) virtual reality training combined with anodal or sham tDCS (Grecco et al., 2015).

These trials lasted 7 weeks, comprising a baseline assessment (1 week prior to the intervention), 2 weeks of intervention (5 sessions per week), a post-intervention assessment (1 week after intervention) and a follow-up assessment (4 weeks after the end of the intervention). The gait training and tDCS started simultaneously and lasted 20 min. Treadmill training was performed on the Inbramed treadmill (Millenium ATL, Brazil) without body weight support. Training speed was 80% of the maximum speed reached during the baseline exercise test. The training speed was gradually increased after the first 2 min and slowly decreased in the last 2 min of treadmill training. Gait training with virtual reality was performed using the Kinect program (Xbox 360, Microsoft, WA, USA). The activity selected (your shape: fitness evolved 2012 run the world) consisted of walking for 20 min in virtual environments, simulating tourist destinations around the world. This game included speed targets—the participants were required to walk with slow and fast paces in random periods determined by the game. Visual and audio feedback was provided when the activity was not performed properly in the virtual reality environment (for further details see Grecco et al., 2014, 2015). It is important to report that treadmill training achieved better results than the virtual reality training on the 6MWT, walking velocity and the gait profile score in both the active and sham tDCS groups (*p* < 0.05 for all analyses).

The tDCS montage was as follows: anodal electrode positioned over the primary motor cortex (between Cz and C3 or C4 positions, following the 10–20 International Electroencephalogram (EEG) System; Homan et al., 1987); and cathode over the supraorbital region on the contralateral side. In children with diparetic CP, the anodal electrode was positioned over the primary motor cortex contralateral to the lower limb with greater motor impairment. For the patients with hemiparetic CP, stimulation was standardized over the affected hemisphere. In the active groups, stimulation at a current intensity of 1 mA was applied for 20 min simultaneously to gait training. For the sham intervention, the device was switched on for 30 s, giving the children the initial sensation of the stimulation, but no current was delivered during the rest of the time.

### Motor Outcomes

All motor outcomes were measured 1 week before the beginning of the intervention (pre-intervention), 1 week after the end of the intervention (post-intervention) and 1 month after the end of the intervention (follow-up). The outcome parameters were absolute changes having occurred during the intervention, considering the post-intervention effect (post minus pre-intervention values) and follow-up effect (follow-up minus pre-intervention values). The following four motor parameters were employed:

– The 6MWT quantifies functional mobility based on the distance in meters covered in 6 min (Borg, 1982). The 6MWT was chosen as primary outcome, since this is a validated test for children with CP and an important quantitative variable of functional gait (Maher et al., 2008).
– Dimension E of the gross motor function measure (GMFM-88) allows a quantitative assessment of walking, running and jumping activities (Russell et al., 2000).
– Gait speed (mean velocity of progression, m/s) was documented using a three-dimensional gait analysis test.
– The gait profile score is based on gait analysis output data. This index was calculated according to the procedure implemented by Baker et al. (2009). It represents the root mean square (RMS) difference between a particular gait trial and averaged data from individuals with no gait pathology. This parameter summarizes the global deviation in the kinematic gait data relative to normative data. The overall gait profile score is based upon gait variable scores that are clinically important kinematic parameters (pelvic anterior/posterior, pelvic up/down obliquity, left-side rotation, hip flexion, abduction, internal rotation, knee flexion, dorsiflexion and foot progression for the left and right sides). In the analysis, a gait profile score was determined for each side based on all nine gait variable scores. A higher gait profile score value denotes a less physiological gait pattern. In the literature, the gait profile score has been used to quantify gait alterations in different adverse health conditions in children and adults (Baker et al., 2009; Cimolin and Galli, 2014).

Since there is no accepted standardization regarding a clinically relevant improvement in the motor outcomes used in the present study (distance traveled on the 6MWT, score on dimension E of the GMFM and gait profile score) for children with spastic CP, a minimum increase of 30% was considered for these variables in the post-intervention and follow-up evaluations (Bartels et al., 2013).

### Neurophysiologic and Neuroanatomic Outcomes

Responses to stimuli applied to the motor cortices were recorded in the quadriceps muscle contralateral to the stimulated side, with two electrodes placed midway between the iliac crest and the lateral joint line of the knee to record vastus lateralis activity (the ground electrode was placed on the contralateral patella). We chose to use the MEP in the quadriceps muscle as this is a gait training study. These measures were performed for the right and left motor cortex. The resting motor threshold (rMT) was evaluated with muscles at rest and measured in each region assessed using five transcranial magnetic pulses for each 2% increment of stimulator output intensity. The children were seated and instructed to remain relaxed without performing muscle contractions of the lower limbs. The vertex was identified and TMS pulses were made 1 cm anterior to 3 cm posterior to the vertex as well as 2 cm over the left and right motor cortices. The MT was defined as the minimum intensity that generates an MEP of at least 100 μV of amplitude in three of five stimuli. TMS was set to an intensity of 110% of the rMT. MEP responses were filtered and amplified using surface electromyography (1000× gain, band-pass filter 20–400 Hz). The signals were processed through offline analysis of the MEP amplitudes. Ten individual MEP measures were recorded and the mean was used for the statistical analysis. The MEP evaluations were performed before and after the interventions as well as during the follow-up evaluation. The MEP was considered absent when there was no response at a stimulator output of 100%.

The children were classified into two groups according to clinical radiological parameters. The cortical group had injuries involving primary motor cortex that could extend to the underlying white matter; and subcortical group had deeper injuries of the internal capsule (excluding the cerebral cortex, brainstem and cerebellum). Radiological classification involved structural magnetic ressonance image (MRI) performed up to 1 year before the onset of the intervention.

The present study was approved by the Human Research Ethics Committee of Nove de Julho University, Brazil (Institutional Review Board (IRB) number: 69803/2012) and it was conducted in accordance with the ethical standards laid down in the Declaration of Helsinki. Written informed consent was obtained from the parents or legal guardians.

## Statistical Analysis

The aim of this exploratory study was to identify the clinical and neurophysiologic characteristics associated with a greater effect of active tDCS in children with CP. Thus, logistic regression models were performed using the responsiveness to the intervention (yes/no) as the outcome (dependent variable). Four outcomes were considered: 6MWT, gait velocity, gait profile score and GMFM. Thus, separate models were run. Responsiveness was defined as a 30% increase in the child’s performance in these outcomes in comparison to baseline. The predictor (independent) variables were age (years), sex (male/female), level of gross motor function (GMFCS levels), topography of the motor impairment (hemiparesis/diparesis), MEP presence, MEP amplitude at baseline evaluation and location of the injury (cortical/subcortical). Univariate logistic regressions were performed for each variable. Based on these initial analyses, predictors associated with outcome at a *p*-value less than or equal to 0.05 were used in the multivariate model.

In addition, we performed analyses of variance (ANOVA) with the Bonferroni *post hoc* test to compare the effects of MEP (present or absent) and injuried area (cortical or subcortical) on the main outcome variables. Correction for multiple comparisons was employed for each variable, resulting in alpha level of 0.0125.

## Results

Fifty-six cases presented the necessary demographic and clinical data to be included in the analysis. All children had motor impairment secondary to injuries of the pyramidal system, with non-progressive lesions having occurred prior to 2 years of age. Thirty-three cases received active tDCS intervention (23 with treadmill training and 10 with virtual reality training) and 23 subjects received sham tDCS intervention (13 with treadmill training and 10 with virtual reality training). Five children had spastic hemiparesis and 51 had spastic diparesis. Mean age at baseline was 8.2 (±1.6) years. There was a significant difference between children with presence or absence of MEP (*p* = 0.04) and cortical or subcortical (sham tDCS groups; *p* = 0.03) regarding gait speed. There were no other significant differences between cortical and subcortical groups or the presence/absence of MEP for other clinical and demographic variables at the baseline assessment (Table 1).

**Table 1:** **Clinical and demographic variables according to injured area (cortical and subcortical) and presence or absence of motor evoked potential (MEP) at baseline assessment**.

	Injury location			Motor evoked potential	
	Cortical	Subcortical	*p*	Present	Absent	*p*
*N*	23	33	–	29	27	–
Age (years)*	8.1 (1.7)	7.5 (1.9)	0.48	8.0 (2.0)	7.8 (2.1)	0.71
Active tDCS	7.3 (1.6)	7.1 (1.1)	0.23	7.6 (1.6)	7.9 (1.9)	0.75
Sham tDCS	7.5 (1.8)	7.8 (1.1)	0.76	8.1 (1.3)	7.6 (2.2)	0.81
GMFCS (I/II/III)**	1/10/12	4/13/16	0.51	3/14/12	2/9/16	0.46
Active tDCS	0/5/5	3/10/10	0.48	1//10/8	1/4/9	0.47
Sham tDCS	1/5/7	1/3/6	0.91	2/4/4	1/5/7	0.94
Hemiparetic/Diparetic**	2/21	3/30	0.92	4/25	1/26	0.80
Active tDCS	1/9	2/21	0.40	3/16	1/13	0.41
Sham tDCS	1/12	1/9	0.84	1/9	0/13	0.37
6MWT (m)*	252.4 (62.7)	241.4 (70.2)	0.54	261.3 (98.4)	249.9 (80.2)	0.45
Active tDCS	242.3 (25.1)	250.5 (57.8)	0.98	261.2 (20.6)	242.3 (25.1)	0.30
Sham tDCS	227.9 (40.8)	251.0 (49.9)	0.06	249.4 (43.3)	227.7 (40.8)	0.25
Gait speed (m/s)*	0.58 (0.17)	0.55 (0.12)	1.0	0.61 (0.13)	0.54 (0.12)	0.04
Active tDCS	0.54 (0.07)	0.57 (0.05)	0.53	0.55 (0.06)	0.54 (0.007)	0.05
Sham tDCS	0.51 (0.16)	0.48 (0.11)	0.03	0.48 (0.07)	0.48 (0.06)	0.08
Gait profile score (°)*	11.3 (2.1)	12.1 (2.9)	0.23	11.9 (3.1)	11.2 (1.7)	0.30
Active tDCS	11.8 (0.6)	11.5 (1.0)	0.06	11.2 (0.5)	11.2 (0.6)	0.28
Sham tDCS	11.3 (0.6)	11.3 (1.0)	0.61	10.9 (0.6)	10.9 (0.7)	0.33
MEP (mV)*	1.5 (0.5)	1.6 (0.4)	0.42	1.6 (0.7)	–	–
Active tDCS	0.9 (0.6)	0.8 (0.7)	0.78	1.3 (0.2)	–	–
Sham tDCS	0.7 (0.4)	0.7 (0.6)	0.93	1.2 (0.3)	–	–
Active tDCS (%)	10 (43.7%)	23 (69.6%)		19 (65.5%)	14 (51.8%)	

Legend: GMFCS, gross motor function classification system; tDCS, transcranial direct current stimulation; 6MWT (m), six-minute walk test; MEP, motor evoked potential. *Data expressed as mean and standard deviation. Analyses performed with unpaired t-test. **Numbers indicate frequency (n) of children in each group. Analyses performed with Chi-square.

The regression models were performed considering all clinical, functional, neurophysiologic and neuroanatomic results. The analyses demonstrated no significant interaction between clinically relevant improvements in the outcomes (minimum increase of 30% on the 6MWT, gait speed, gait profile score and dimension E of the GMFM) and clinical or demographic variables such as age, sex, motor function level (levels I, II or III of the GMFCS) or type of motor impairment (hemiparesis or diparesis).

The univariate logistic analysis considering only the children who received active tDCS combined with gait training revealed two predictors significantly associated with the responsiveness to the intervention: MEP present during the initial evaluation and location of the injury (Table 2). The presence of MEP was significantly associated with responsiveness, based on the 6MWT (*p* = 0.003) and gait speed (*p* = 0.028). Considering the results of the follow-up evaluation (1 month after the end of the intervention), a significant association was only found between the presence of MEP and the 6MWT (*p* = 0.010). The analysis also showed that the subcortical injury was a significant predictor of improvements in 6MWT (*p* = 0.004), gait profile score (*p* = 0.013) and gross motor function (*p* = 0.021). Table 3 displays the functional findings considering the presence/absence of MEP and location of the injury (cortical or subcortical).

**Table 2:** **Results of linear logistic regressions in the active and sham tDCS groups**.

		Active tDCS				Sham tDCS	
Variables	Respondent (Yes/No)	B (SE)	Exp (B)	*p*	Respondent (Yes/No)	B (SE)	Exp (B)	*p*
6MWT
MEP present	19/0	2.5 (0.86)	8.9	0.003	5/4	0.9 (0.76)	0.4	0.400
Subcortical injury	18/5	2.6 (0.93)	8.0	0.004	6/4	0.8 (0.38)	0.6	0.664
Gait velocity
MEP present	16/3	1.6 (0.77)	4.8	0.028	3/6	0.8 (0.90)	2.4	0.331
Subcortical injury	10/13	1.1 (0.80)	1.8	0.170	6/4	0.8 (0.76)	0.4	0.384
Gait profile score
MEP present	10/9	1.0 (0.74)	1.9	0.168	4/5	1.7 (1.30)	0.1	0.172
Subcortical injury	17/6	2.1 (0.85)	6.1	0.013	4/6	1.2 (1.12)	0.3	0.281
GMFM dimension E
MEP present	11/8	1.0 (0.73)	2.1	0.147	3/6	2.0 (1.30)	0.1	0.120
Subcortical injury	15/8	1.9 (0.84)	5.3	0.021	7/3	1.2 (0.73)	0.4	0.479

Respondent (Yes/No) shows the frequency of children considered responders to intervention with MEP present (active tDCS: 19 and sham tDCS: 9 children) and subcortical injury (active tDCS: 23 and sham tDCS: 10 children). Legend: SD, standard deviation; B, B coefficient; SE, standard error of B coefficient; Exp (B), measurement of likelihood ratio of association between predictor variable and outcome. *p < 0.05.

**Table 3:** **Mean and SD of results in children with presence and absence of MEP, and subcortical and cortical lesions considering active and sham tDCS**.

	MEP present		MEP absent		Subcortical injury		Cortical injury	
	Active	Sham	Active	Sham	Active	Sham	Active	Sham
6MWT (m)
Pre-intervention	261.2 (20.6)	249.4 (43.3)	242.3 (25.1)	227.7 (40.8)	250.5 (57.8)	251.0 (49.9)	241.5 (28.3)	212.1 (43.9)
Post-intervention	418.2 (60.8)*	310.1 (11.8)	366.9 (41.3)	331.1 (47.1)	393.9 (62.4)^#^	339.0 (54.4)	345.3 (40.5)	317.6 (79.5)
Follow-up	393.8 (58.2)	286.8 (92.2)	356.9 (47.9)	305.3 (50.3)	354.2 (62.1)	322.2 (42.9)	338.4 (53.8)	287.4 (56.3)
Gait speed (m/s)
Pre-intervention	0.55 (0.06)	0.48 (0.07)	0.54 (0.07)	0.48 (0.06)	0.54 (0.11)	0.51 (0.16)	0.57 (0.05)	0.48 (0.11)
Post-intervention	0.82 (0.16)*	0.56 (0.12)	0.59 (0.08)	0.56 (0.12)	0.77 (0.18)^#^	0.62 (0.19)	0.68 (0.11)	0.57 (0.10)
Follow-up	0.71 (0.13)	0.58 (0.06)	0.56 (0.05)	0.58 (0.06)	0.70 (0.13)	0.58 (0.24)	0.63 (0.06)	0.54 (0.14)
Gait profile score (%)
Pre-intervention	11.2 (0.5)	10.9 (0.6)	11.2 (0.6)	10.9 (0.7)	11.8 (0.6)	11.3 (0.6)	11.5 (1.0)	11.3 (1.0)
Post-intervention	8.8 (0.9)*	9.2 (0.8)	9.1 (1.1)	9.1 (0.5)	8.5 (1.2)^#^	9.9 (0.6)	10.4 (1.0)	9.8 (1.4)
Follow-up	9.8 (0.9)	9.7 (1.0)	9.7 (0.2)	9.7 (0.8)	9.4 (1.6)^#^	10.0 (1.1)	10.8 (1.1)	10.3 (1.3)
GMFM-88 (dimension E)
Pre-intervention	53.9 (13.0)	56.7 (8.6)	52.1 (11.2)	48.8 (12.4)	52.0 (11.3)	53.4 (19.9)	51.6 (10.9)	49.8 (13.8)
Post-intervention	69.4 (14.4)	76.3 (11.6)	69.3 (9.7)	62.8 (12.6)	68.8 (13.7)^#^	66.4 (10.5)	63.7 (14.3)	61.6 (16.8)
Follow-up	66.2 (15.7)	68.9 (10.6)	64.1 (9.8)	61.1 (14.4)	63.8 (14.1)	63.6 (8.2)	57.1 (12.1)	59.4 (15.9)
MEP (mV)
Pre-intervention	1.3 (0.2)	1.2 (0.3)	–	–	0.9 (0.6)	0.7 (0.4)	0.8 (0.7)	0.7 (0.6)
Post-intervention	2.3 (0.3)	1.5 (0.4)	–	–	1.5 (1.0)	1.0 (0.9)	1.4 (1.3)	0.9 (0.8)
Follow-up	1.6 (0.4)	1.2 (0.5)	–	–	1.0 (0.8)	0.8 (0.5)	1.0 (0.9)	0.7 (0.6)
Resting motor threshold (rMT, %)	57.9 (11.1)	60.1 (13.2)	–	–	52.6 (12.6)	54.3 (13.5)	58.8 (11.8)	57.6 (12.5)

Legend: GMFM, gross motor function measure. Analyses performed using analysis of variance (ANOVA) followed by Bonferroni post hoc test. *p < 0.05: analysis considering the groups: MEP present and active tDCS, MEP present and sham tDCS, MEP absente and active tDCS, MEP absent and sham tDCS. ^#^p < 0.05: analysis considering the groups: subcortical injury and active tDCS, subcortical injury and sham tDCS, cortical injury and active tDCS, cortical injury and sham tDCS.

To determine whether there may have been a confounding effect between the presence of MEP and injury location, both variables were incorporated simultaneously in the logistic regression model. The results of this analysis demonstrated that the presence of MEP remained significantly associated with the results of the 6MWT (*p* = 0.007) and gait speed (*p* = 0.011), and that the subcortical injury remained significantly associated with the gait profile score (*p* = 0.038) and gross motor function (*p* = 0.046).

We also incorporated the type of gait training (treadmill training and virtual reality training) into the regression model to adjust the analysis to the type of training, but no significant changes in the results were found. Then, we tested whether there was an interaction between these two variables and found that the type of formation virtually exerted no alteration in the interaction of these variables. The presence of MEP continued to demonstrate a significant association with 6MWT (*p* = 0.006) and gait speed (*p* = 0.039), and subcortical injury demonstrated a significant association with the gait profile score (*p* = 0.008). However, the association between subcortical injury and gross motor function was not maintained in this analysis (*p* = 0.095).

Multivariate regression analysis was performed with the incorporation of the variable MEP * injury location to determine a possible interaction with the functional outcomes. The analysis demonstrated that a significant interaction was only found with the 6MWT (*p* = 0.001), whereas no significant associations were found with regard to gait speed (*p* = 0.519), gait profile score (*p* = 0.358) or gross motor function (*p* = 0.103).

As the primary objective of the present study was to identify factors predictive of the response to active tDCS, the analyses and discussion focused only on the results obtained through the regression models that incorporated the participants who have received active tDCS. To clarify the findings, however, it should be stressed that the linear regression analyses were performed considering the group of children who received sham tDCS and measures considered possible response factors. There were no significant interactions between MEP and injury location with regard to the variables (Table 2).

**Figure 1:**
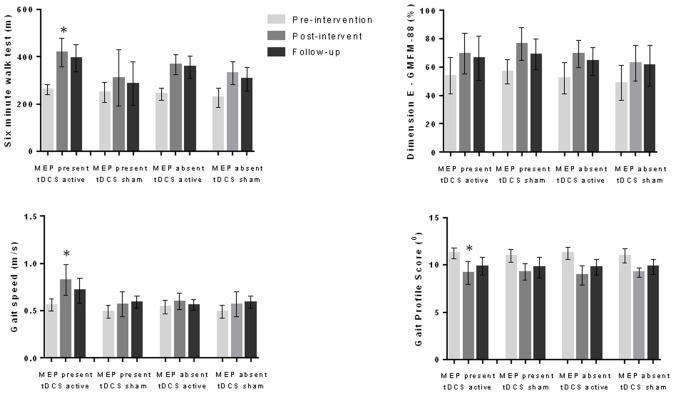
**Data for six-minute walk test (6MWT), gait speed, gait profile score and dimension E of the gross motor function measure (GMFM-88) during pre-intervention, post-intervention and follow-up evaluations considering motor evoked potential (MEP) present and absent as well as active and sham transcranial direct current stimulation (tDCS; 56 children).** Children with MEP present at pre-intervention evaluation and who received active tDCS demonstrated better results at the post-intervention evaluation in comparison to other groups for 6MWT, gait speed and gait profile score. *p < 0.05 analysis of variance (ANOVA) followed by Bonferroni *post hoc* test.

**Figure 2:**
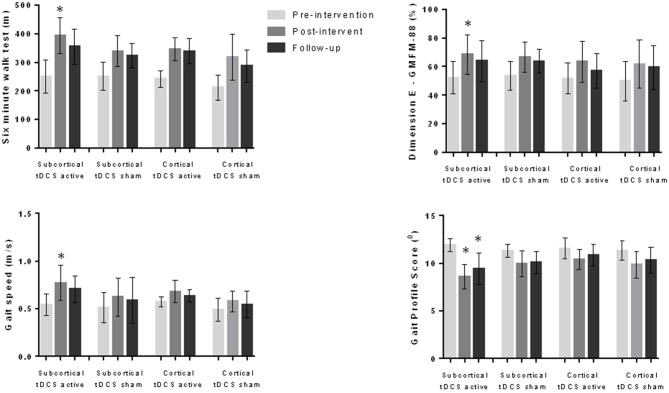
**Data for 6MWT, gait speed, gait profile score and dimension E of the GMFM-88 during pre-intervention, post-intervention and follow-up evaluations considering subcortical and cortical injuries as well as active and sham tDCS groups (56 children).** Children with subcortical injury and who received active tDCS presented better results at the post-intervention evaluation in comparison to other groups for all variables. This group also had better results regarding gait profile score at follow-up evaluation. *p < 0.05 ANOVA followed by Bonferroni *post hoc* test.

We compared the effects on the variables analyzed considering the presence or absence of MEP (MEP present and active tDCS; MEP present and sham tDCS; MEP absent and active tDCS; MEP absent and sham tDCS) and the location of the injury (cortical lesion and active tDCS; cortical lesion and sham tDCS; subcortical lesion and active tDCS; subcortical lesion and sham tDCS). The variance analysis performed considering the four subgroups refering to the MEP demonstrated significant differences in 6MWT (*F*_(11,1)_ = 22.1, *p* < 0.001), gait velocity (*F*_(11,1)_ = 13.0, *p* < 0.001) and gait profile score (*F*_11,1_ = 13.8, *p* < 0.001). The *post hoc* test showed that the subgroup “MEP present and active tDCS” achieved better results than the other groups regarding the 6MWT (*p* < 0.001), gait velocity (*p* < 0.001) and gait profile score (*p* < 0.001) during the post-intervention evaluation (Figure 1). In the analysis considering the four subgroups related to location of the lesion, different effects were found regarding 6MWT (*F*_(11,1)_ = 15.3, *p* < 0.001), gait velocity (*F*_(11,1)_ = 5.6, *p* < 0.001), gait profile score (*F*_(11,1)_ = 5.0, *p* < 0.001) and dimension E of GMFM (*F*_(11,1)_ = 3.4, *p* = 0.003). The *post hoc* test showed that the subgroup “subcortical injury and active tDCS” achieved better results than the other subgroups analyzed regarding 6MWT (*p* < 0.001), gait velocity (*p* = 0.007), gait profile score (*p* = 0.024) and dimension E of GMFM (*p* = 0.002; Figure 2).

## Discussion

Although the effects of tDCS are encouraging, divergent results are often encountered. The clinical predictors of responsiveness to this method are not currently known. Therefore, the major challenge in clinical investigations on noninvasive brain stimulation is to identify biomarkers associated with positive responses. Levels of BDNF in patients with depressive disorders (Fidalgo et al., 2014) and levels of ipsilesional GABA in stroke survivors are biomarkers commonly cited in the literature (O’shea et al., 2014).

Since the primary motor cortex is the most commonly employed electrode montage and considering the modulation of the MEP amplitude, we had predicted that: (1) the benefits of tDCS would be related to the preservation of the underlying primary motor cortex (not affected by the injury); and (2) the presence of MEP, demonstrating the integrity of the corticospinal tract, could also be related to the beneficial effects of anodal tDCS. We hypothesized that tDCS would have a greater effect on children with CP with an intact primary motor cortex to receive anodal tDCS and when an MEP could be produced, as such children would have anatomic and physiologic evidence of their potential to responsiveness to such treatment. The findings demonstrated that the presence of a pre-intervention MEP was significantly associated with children with CP, who responded satisfactorily to gait training combined with anodal tDCS in terms of the distance traveled on 6MWT and gait speed, whereas the subcortical location of the injury demonstrated a significant association with the kinematic gait pattern and gross motor function.

Different neurophysiologic mechanisms are involved in the physiopathology of CP. Spastic CP is secondary to a pyramidal injury, compromising voluntary motor control with a reduction in cortical activity in motor areas, leading to a reduction in up-down muscle control (Burton et al., 2009; Kurz and Wilson, 2011; Pitcher et al., 2012). To evaluate the MEP in previous studies by our research group, we quantified electromyographic activity in the quadriceps muscles, which is important to the prognosis of standing and walking. Therefore, it is more appropriate to analyze a muscle directly related to the trained motor activity. We recognize that the cortical representation of the quadriceps muscle is more complex due to its location in comparison to an upper limb muscle. However, we believe that the use of the MEP of an upper limb muscle would not adequately represent this study goals. The presence of MEP can be understood as the integrity of the corticospinal tract to generate muscle activity or control muscle movement, therefore being considered a reliable predictor of clinical responsiveness. Thus, the use of anodal tDCS over the primary motor cortex likely facilitated the activity of this cortical area during gait training and consequently optimized the progress of motor training.

The literature suggests that an absent MEP may be associated with a poor prognosis in adult stroke survivors with hemiparesis (Van Kuijk et al., 2009; Lai et al., 2015). Although no studies were encountered on this aspect in children with CP, we found that the children responded to motor rehabilitation equally well with or without MEP. It should be stressed that children who received either active or sham stimulation responded to gait training, as described in previous studies. However, those who received active stimulation demonstrated greater effects in comparison to those who underwent sham stimulation. Thus, one may suggest that a single-pulse TMS can be used to identify subjects who will be respondent to tDCS by the presence or absence of a MEP, but not those who will be responsive to motor training. This finding indicates the specificity of tDCS affecting the corticospinal system to exert a supplementary benefit.

We believe that this result may be related to the adaptation process following a brain injury. Previous studies have demonstrated that a significant number of children with hemiparetic CP have cortical motor representations ipsilateral to movement (Mackey et al., 2014; Pihko et al., 2014). In cases of diparesis, the information is not yet fully clarified and there may be ipsilateral representations or even bilateral representations of movement (Kesar et al., 2012). Neurophysiologic studies have demonstrated that ipsilateral representations are strongly associated with greater motor impairment and there is no specific information on the relationship between cortical representations and the responsiveness to motor rehabilitation (Holmström et al., 2010; Van de Winckel et al., 2013; Mackey et al., 2014).

The present study did not include an evaluation of the area referring to the cortical motor representation (ipsilateral or contralateral to movement). We evaluated the amplitude of the MEP of the primary motor cortex. As all children exhibited active movement of quadriceps muscle (knee extension movement with sufficient force to overcome gravity), some area of the brain was apparently responsible for the control of this movement. Thus, this aspect may be considered a limitation of the present study. Administering anodal stimulation over most affected primary motor cortex without being sure that the area was responsible for the motor control of the most affected lower limb (especially in children with hemiparesis, since the unaffected brain hemisphere may be responsible for the control of both hemibodies), we assume the risk of tDCS not generating the desired modulatory effects and not optimizing the benefits of gait training. However, it should be emphasized that our previous randomized controlled studies, from which the data for the present investigation were extracted, demonstrated significant improvements in the groups that received active tDCS combined with gait training, even when tDCS was administered over the more affected primary cortex and with anode positioned between Cz and C3 or C4. This suggests that the montage was beneficial to optimizing motor gains and contributed to the maintenance of the results 1 month following the end of the interventions. A hypothesis for this finding is based on modeling studies indicating that the current and effects are distributed between the anode and cathode rather than merely beneath the anode. Thus, the area responsible for the lower limbs may have been involved in the present investigation.

We believe that the evaluation of the complete motor map, with the administration of TMS pulses in the motor cortex bilaterally during bilateral electromyographic readings could clarify the influence of cortical representation and the presence of the MEP on the effect of the intervention in children with CP. The motor cortical map enables identification of the brain area responsible for controlling movements (that motor training could improve) and a more precise analysis of the corticospinal tract integrity. Thus, it could be possible to determine the target area for noninvasive brain stimulation more precisely, thereby increasing the efficiency of this technique. If this procedure were performed prior to or following the motor intervention process, the results could assist in clarifying cerebral motor adaptations in CP as well as provide information on the adaptation process of the brain during physical rehabilitation.

The effect of active tDCS on 6MWT was significantly greater among children with CP with a present MEP in comparison to those without the presence of this physiological aspect during the pre-intervention evaluation. This finding, together with the results of the regression analyses demonstrating a significant interaction between MEP and 6MWT, suggests that MEP may be a prerequisite for complementary treatment with tDCS. The results of the statistical analyses make sense and lend support to our hypotheses. To obtain the clinical effects of tDCS, it is important to have connectivity between the stimulated cortical area and structures of the neural motor system responsible for execution of the motor activity. Therefore, careful selection of target areas for noninvasive brain stimulation is of extreme importance. Apparently, the determination of such area based exclusively on the location of the injury and merely by the aim of either facilitating or inhibiting a given area is insufficient for adequate therapeutic planning. Moreover, the 10–20 EEG system commonly employed in clinical trials may not be the best option for locating the target area for stimulation in children with CP. However, these suppositions need to be tested in future studies.

Previous randomized controlled studies conducted by our research group, from which the data for the present investigation were extracted, demonstrated significant improvements in groups that received active tDCS combined with gait training, even when tDCS was administered over the more affected primary motor cortex. Thus, one could infer that this montage is beneficial to optimizing motor gains and contributes to the maintenance of such gains 1 month after the end of the intervention.

The motor prognosis of children with CP is generally based on different aspects related to the injury such as its location and extent. For a long time, the size of injury was considered the main factor associated with the development of satisfactory or unsatisfactory motor development. Currently, studies have demonstrated that injury size is not necessarily the major aspect governing the magnitude of motor impairment. Neurophysiologic adaptations secondary to the injury may exert an important influence on the functional prognosis. The literature offers no further information on the impact of cortical-subcortical injuries in comparison to injuries that exclusively affect cortical areas regarding neurophysiologic adaptations in this population.

The classification of the injury (cortical and subcortical) has proven to be extremely important in the field of noninvasive brain stimulation. During tDCS, there is significant dispersal of current and only a small amount reaches out cortical areas. The subcortical effects of tDCS are secondary to alterations in the cortical excitability generated by the current that reaches the cortex. Children with subcortical injuries who received active tDCS achieved better results on gait profile score and gross motor function than those with cortical injuries who received active stimulation. However, acquisition of motor functions following an injury to the cortex is possible. As shown in our previous studies, children with CP demonstrate improvement of the variables studied after gait training, but this study showed that the effects of gait training were optimized with active tDCS, especially in children with subcortical lesions.

Physiotherapists observe important motor gain in their patients every day. Motor acquisitions are often achieved through numerous postural compensations in the medium and long terms, which can lead to additional problems for the patient. Adequate motor control during the execution of a movement requires the precise activation of superior neurological systems. Although the present findings may have been influenced by other factors and it is not possible to perform an exclusive analysis on the role of the subcortical injury regarding movement quality, we believe that future studies with the aim of more adequately clarifying the impact of cortical and subcortical injuries on movement quality in children with CP are of extreme importance to the field. The present study was unable to prove the hypothesis that cortical damage compromises movement quality more than subcortical damage, but could be used for the development of future studies addressing this issue (Rahman et al., 2015).

Adults with hemiparesis following a cortical injury demonstrate poor results with regard to noninvasive brain stimulation in comparison to those with subcortical injuries (Reynolds et al., 2014). The present results demonstrate that the MEP * injury location interaction is a predictor of the responsiveness to the intervention regarding the 6MWT assessment. It is likely that the heterogeneity in the results of the group with cortical injuries is related to the amount of preserved nerve tissue over the area to which tDCS was administered. Thus, further studies should be developed that include the specific quantification of damage to the corticospinal tract with the use of tractography, for example. The results obtained with this method could clarify the relationship between the extent of the motor tract injury and the cortical effects of tDCS.

The present investigation is an exploratory study that presents a critical discussion regarding the importance of understanding the influence of neurophysiologic and neuroanatomic biomarkers on the results obtained with the combination of tDCS and motor training in children with CP. The findings demonstrate that the presence of MEP is associated with functional measures such as 6MWT and gait speed, whereas the subcortical injury is associated with more specific variables (e.g., gait kinematics). There is a need for further studies performing a more in-depth exploration through controlled clinical trials on the influence of MEP, the cortical representation of movement and the location of injury regarding the responsiveness to the use of tDCS; and also seeking to understand the influence of these measures on the motor prognosis of children with CP, and jump thick in activities such as walking, running, movement (cinada to gait training for the 6MWT).

The main limitation of the present study was the use of a secondary analysis of previous trials data. Therefore, no specific sample size calculation was performed. Regarding the primary outcome (6MWT) in the analysis comparing children with cortical or subcortical injuries, 74 subjects per group would be necessary to possibly demonstrate an effective influence of injury location on gait training. However, this is an innovative, pioneering study that assessed the influence of neurophysiologic and anatomic parameters on the effects of gait training combined with tDCS. Additional investigations may provide specific information to clarify the interaction between these aspects and neural motor recovery in patients with CP. Other possible predictive factors should be investigated in future trials such as specific location and extent of injury (including an analysis of periventricular lesions), and motor cortical adaptation (motor cortical representation—unilateral, bilateral or contralateral and pattern of cortical activation during movement through functional magnetic resonance imaging (fMRI) and tractography). Studies on the effects of tDCS and its parameters for use in children with CP are still in early stages and many questions need to be clarified before the technique can be considered effective at optimizing the physical rehabilitation of these children.

## Author Contributions

LACG, DJE and FF designed research; LACG, NdACD, CC, NZ and CSO performed research; LACG, MG, DJE and FF analyzed data; LACG, DJE and FF wrote the article.

## Conflict of Interest Statement

The authors declare that the research was conducted in the absence of any commercial or financial relationships that could be construed as a potential conflict of interest.

## Abbreviations

EEG: electroencephalogram
IRB: Institutional Review Board
GMFM: gross motor function measure
GMFCS: gross motor function classification system
MRI: magnetic ressonance image
MEP: motor evoked potential
RMS: root mean square
tDCS: transcranial direct current stimulation
TMS: transcranial magnetic stimulation
6MWT: six-minute walk test.
